# Microwave-Assisted Synthesis of Nitrogen-Doped Carbon Dots for Sensitive Fluorescence-Based Urea Detection in Soil

**DOI:** 10.1007/s10895-025-04399-4

**Published:** 2025-06-24

**Authors:** Numy Eranikkal, K. R. Riyamol, Hajera Shafaf, Mohammad Nawshad, Muni Raj Maurya, Anwarul Hasan, Probir Das, Kishor Kumar Sadasivuni

**Affiliations:** 1 Center for Advanced Materials, Qatar University, P.O. Box 2713, Doha, Qatar; 2 Department of Mechanical and Industrial Engineering, Qatar University, P.O. Box 2713, Doha, Qatar; 3 College of Arts and Sciences, Qatar University, Doha, P.O. Box 2713, Qatar

**Keywords:** CDs, Microwave, Urea, Sensing, Fluorescence probe

## Abstract

This study presents a rapid, green, and highly sensitive fluorescence-based method for detecting urea in soil using nitrogen-doped carbon dots (CDs) synthesized via a microwave-assisted process. Citric acid and urea were used as carbon and nitrogen precursors, respectively, with optimal synthesis achieved at a 1:1 weight ratio and 9 min of microwave irradiation. The structural properties of nitrogen-doped carbon dots (CDs) were studied using X-ray Diffraction (XRD). Transmission Electron Microscopy (TEM) technique was used to observe the shape and size of the CDs, providing insight into their morphology. To understand the chemical composition, bonding states, and surface functionalities, X-ray Photoelectron Spectroscopy (XPS) and Fourier Transform Infrared Spectroscopy (FTIR) analyses were carried out. The performance of the nitrogen-doped-CDs in detecting urea, including their sensitivity and selectivity, was evaluated using fluorescence spectroscopy. The resulting CDs exhibited enhanced fluorescence properties and a limit of detection (LOD) as low as 143 mg/gm. The method demonstrated high selectivity toward urea even in the presence of interfering metal ions, and its effectiveness was validated in soil samples under varying pH conditions. This approach provides a cost-effective, scalable, and environmentally friendly solution for real-time monitoring of soil nutrients, supporting sustainable agricultural practices through improved nitrogen management.

## Introduction

Urea [(CO(NH₂) ₂)] is recognized as the predominant nitrogenous fertilizer utilized worldwide, constituting approximately fifty per cent of the aggregate nitrogen fertilizers employed in agricultural practices [1]. Its substantial nitrogen concentration (46%), cost-effectiveness, and straightforward application make it essential for enhancing soil fertility and agricultural productivity, which are key for ensuring global food security [2]. However, inappropriate urea utilization can lead to considerable environmental ramifications [1]. In the ground, urea undergoes hydrolysis facilitated by urease, quickly converting into ammonia (NH₃) and carbon dioxide (CO₂), resulting in nitrogen depletion and ammonia escaping into the atmosphere, exacerbating pollution and reducing nitrogen utilization efficiency [3]. Using urea also boosts nitrous oxide (N₂O) emissions, a significant greenhouse gas with a global warming potential three hundred times greater than CO₂ [2]. Additionally, runoff from fields treated with urea can instigate eutrophication in aquatic ecosystems, presenting severe risks to biodiversity and water quality [4].

Numerous analytical methodologies have been established to accurately quantify urea within intricate matrices such as soil, aqueous environments, and biological fluids. In traditional practices, titration and colorimetric assays are employed with the highest regularity [5]. Although these techniques are relatively straightforward and economically viable, they are characterized by substantial time demands and labor intensity, making them unsuitable for real-time or field-based applications [6].

When urea reacts with certain reagents in colorimetric assays, as demonstrated by the diacetyl monoxime approach, a color modification occurs that can be quantitatively determined via spectrophotometric evaluation [5]. These assays are extensively utilized owing to their cost-effectiveness and simple procedural guidelines. Nonetheless, they frequently necessitate the use of hazardous reagents and exhibit restricted sensitivity, particularly at diminished urea concentrations [4]. Furthermore, interference from soil constituents such as organic matter and salts may undermine the precision of these methodologies in environmental contexts [5].

Recent innovations have demonstrated enzyme-based biosensors as a viable alternative for detecting urea. Employing urease enzymes, these sensors facilitate the breakdown of urea, resulting in observable changes in pH, conductivity, or electrochemical metrics [7]. Potentiometric biosensors, which measure pH variations induced by ammonia generation, have shown remarkable specificity for urea, rendering them appropriate for both clinical and agricultural applications [8]. However, enzyme-based biosensors encounter several obstacles, including limited stability, sensitivity to fluctuating environmental conditions, and elevated production costs. The instability of urease, along with its vulnerability to denaturation under extreme circumstances, further constrains the practicality of these sensors in field environments [7, 9].

Electrochemical sensors are another advanced method for urea detection. To increase sensitivity and selectivity, these devices are often equipped with modified electrodes, such as those coated in silver nanoparticles (AgNPs) [10]. Electrochemical techniques that use amperometric and potentiometric instruments produce quick and portable solutions that are perfect for in-field analyses [10]. Despite these advantages, obstacles such as electrode contamination, expensive materials, and a limited detection range in difficult matrices like dirt prevent their broader application [11].

New methods based on fluorescence-based sensing offer a very beneficial substitute for urea detection [12]. Much research has been done on fluorescent probes because of their exceptional sensitivity, quick response, and environmental resilience, such as carbon dots (CDs) and quantum dots [13]. These techniques rely on the probe’s optical properties, which cause them to release fluorescence signals when they come into contact with particular analytes, including urea [14, 15]. CDs have grabbed a lot of attention because of their economic feasibility, low toxicity, and tunable photoluminescence and are, therefore, suitable for use in soil analysis [16, 17]. For example, urea may be detected selectively and sensitively in complex environmental matrices thanks to the increased fluorescence properties of CDs made from citric acid and urea [18, 19].

CDs have been created using various methods to detect pesticides and fertilizers, including hydrothermal methods, electrochemical processes, and chemical vapor deposition techniques. Hydrothermal synthesis entails high-temperature and high-pressure reactions, yielding CDs with adjustable properties; however, this approach necessitates prolonged reaction durations and substantial energy consumption [20]. Electrochemical synthesis permits meticulous control over surface functionalization, enhancing selectivity for agrochemical detection; nevertheless, it requires intricate instrumentation and expensive electrodes [21]. CVD can produce high-purity CDs with superior crystallinity; however, it is constrained by the need for costly apparatus and inert atmospheres [22]. In contrast, this investigation adopts a green, rapid, and energy-efficient microwave-assisted synthesis approach, which enables the synthesis of nitrogen-doped CDs in merely 9 min, thereby significantly abbreviating reaction times compared to conventional methodologies. This technique is congruent with the principles of green chemistry as it minimizes solvent utilization and eliminates hazardous substances while enhancing fluorescence sensitivity to detect urea [23]. CDs have demonstrated efficacy in detecting pesticides such as Carbendazim [23], Hexaconazole Fungicide [22], Triazophos [20], Cypermethrin, and Lambda-Cyhalothrin [21]. Incorporating nitrogen doping from urea augments selectivity, rendering this method exceptionally effective for precision agriculture applications. In contrast to traditional methodologies, microwave-assisted synthesis guarantees scalability, cost-effectiveness, and enhanced optical characteristics, thereby offering an innovative and practical solution for monitoring soil nutrients.

CDs are primarily prepared with microwave assistance, a revolutionary synthesis technique that has drastically changed the field of luminous nanomaterials [24]. Unlike conventional heating methods, microwave radiation enables rapid and uniform heating, speeds up reaction kinetics, reduces synthesis times, and improves material properties [24, 25]. Because it uses fewer solvents and less energy, this method is in line with the ideas of green chemistry and, therefore, economically and environmentally viable [26, 27]. The efficiency of microwave-assisted synthesis in producing nitrogen-doped CDs with improved optical and structural characteristics has been confirmed by recent studies, opening the door for their use in fluorescence-based sensing platforms [15, 28].

Citric acid, a naturally occurring organic compound, has gained prominence as a precursor for CDs synthesis owing to its multifunctional characteristics and widespread availability [17, 29, 30]. The presence of carboxyl and hydroxyl functional groups in citric acid promotes the formation of stable nanostructures with substantial fluorescence [19], while its compatibility with microwave-assisted synthesis guarantees the rapid and efficient generation of CDs [18]. Utilizing citric acid with urea is vital for synthesizing nitrogen-doped CDs, characterized by their remarkable fluorescence and elevated selectivity towards urea detection [16, 28]. The optimization of reaction parameters, including the citric acid-to-urea ratio and microwave power, facilitates meticulous control over the dimensions, surface characteristics, and quantum yield of the resultant CDs, thereby augmenting their efficacy as urea sensors [24, 27].

The current investigation intends to establish an effective fluorescence-based methodology for urea detection in soil. It employs microwave-assisted synthesis of nitrogen-doped CDs derived from citric acid and urea. By integrating the sensitivity inherent to fluorescence detection with microwave synthesis’s scalability and environmental sustainability, this study aims to mitigate the shortcomings of prevailing urea detection methodologies. The specific aims encompass optimizing synthesis parameters for high-performance CDs and assessing their fluorescence response to varying urea concentrations in soil.

## Materials and Methods

### Chemicals and Reagents

Citric acid (CA) (HOC(COOH)(CH2COOH)_2_, ≥ 99.5%) and urea (NH_2_CONH_2_, ≥ 99%) were procured from Sigma-Aldrich (United States). Sodium hydroxide (NaOH) pellets were purchased from Fischer Chemicals. Hydrochloric acid (HCl, 37%) was purchased from VWR BDH chemicals. The chemicals employed in the study are of reagent grade and were not subjected to any additional purification before their application. Experimental procedures were carried out using Millipore Milli-Q double-distilled water.

### Optimization of Urea and Citric Acid for the Synthesis of Carbon Dots

**Fig. 1:**
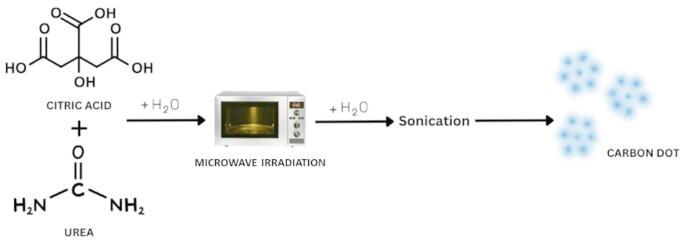
Schematic representation of the synthesis of CDs

Fig. 1 shows the schematic representation of the methodology adopted for synthesizing CDs. The amount of urea for synthesizing CDs was optimized by adding various urea concentrations (1, 3, 5, 7, 9 mg) to 10 mg of citric acid. The mixture was microwaved at 680 watts by adding 10 ml of deionized water. The semi-solid substance obtained after the microwave process was diluted with 5 ml of DI water and sonicated. The different samples were then subjected to check UV absorbance and fluorescence. The same procedure was followed for the optimization of citric acid. Different concentrations of citric acid (1, 3, 5, 7, 9 mg) were added to the optimized urea concentration. After the microwave process, the samples were subjected to check fluorescence.

### Optimization of Microwave Time

The microwave time was optimised by examining the fluorescence obtained by the citric acid and urea CDs at varying times (5, 6, 7, 8, and 9 min). After microwaving at each decided time, the fluorescence was measured using a spectrofluorometer.

### Purification of Synthesized Carbon Dots

The viscous liquid carbon dots formed were further heated until all volatile and water components evaporated. The solid mass obtained is then washed with acetone to purify the carbon dots and remove excess organic residues. Sonication is done to enhance the purification process. The solution was filtered using Whatman filter paper, which separated solid carbon dots from the acetone solution. Several rounds of the same method are performed to ensure the complete removal of organic impurities. The dried CDs were then re-suspended with distilled water for further use.

### Detection of Urea in the Soil Sample

Urea at concentrations of 1, 3, 5, and 10 mg was blended with 10 mg of soil. Subsequently, the 10 ml deionized water was added, and 5 mg of citric acid was incorporated into the solution. The resultant solutions were subjected to a microwave treatment for 9 min at a power output of 680 watts. After the microwave exposure, the solutions received an extra 5 ml of deionized water for dilution. The samples were subsequently subjected to fluorescence analysis utilizing a spectrofluorometer.

### Effect of pH

Five discrete soil samples, each with a mass of 10 mg, were prepared, followed by adding 5 mg of urea into each specimen. The specimen’s pH levels were carefully modified to readings of 3, 5, 7, 9, and 11 by using appropriate pH adjustment solutions (NaOH, HCL). Following the pH modifications, an additional 5 mg of citric acid was introduced into each specimen to ensure uniform conditions and to promote the biochemical reactions. The resulting specimens were subsequently subjected to fluorescence analysis to evaluate the impact of pH fluctuations on their fluorescence properties.

### Characterization Techniques

The structural characteristics of the samples were analyzed using an X-ray diffractometer (X’PERT-Pro MPD, PANalytical Co., Almelo, Netherlands) and Transmission electron microscopy (TEM, JEM2002-FS, JEOL). The formation of carbon quantum dots (CQDs) was verified using a Thermo Nicolet Nexus 670 FTIR spectrometer employing KBr pellets. The microstructural and topographical studies were done by doing profilometry using an OLYMPUS DSX1000 optical profilometry. The microstructural aspects of the sample were viewed using Scanning electron microscopy (JEOL, JCM6000). Elemental surface analysis was done using X-ray photoelectron Spectrometer (Axis ultra DLD). A spectrofluorophotometer was employed to assess fluorescence emission spectra at an excitation wavelength of 330 nm. In opposition, a UV-visible spectrophotometer was applied to record the absorption spectra of the CQD solutions in an aqueous medium.

## Result and Discussion

### Optimization of Citric Acid and Urea

**Fig. 2:**
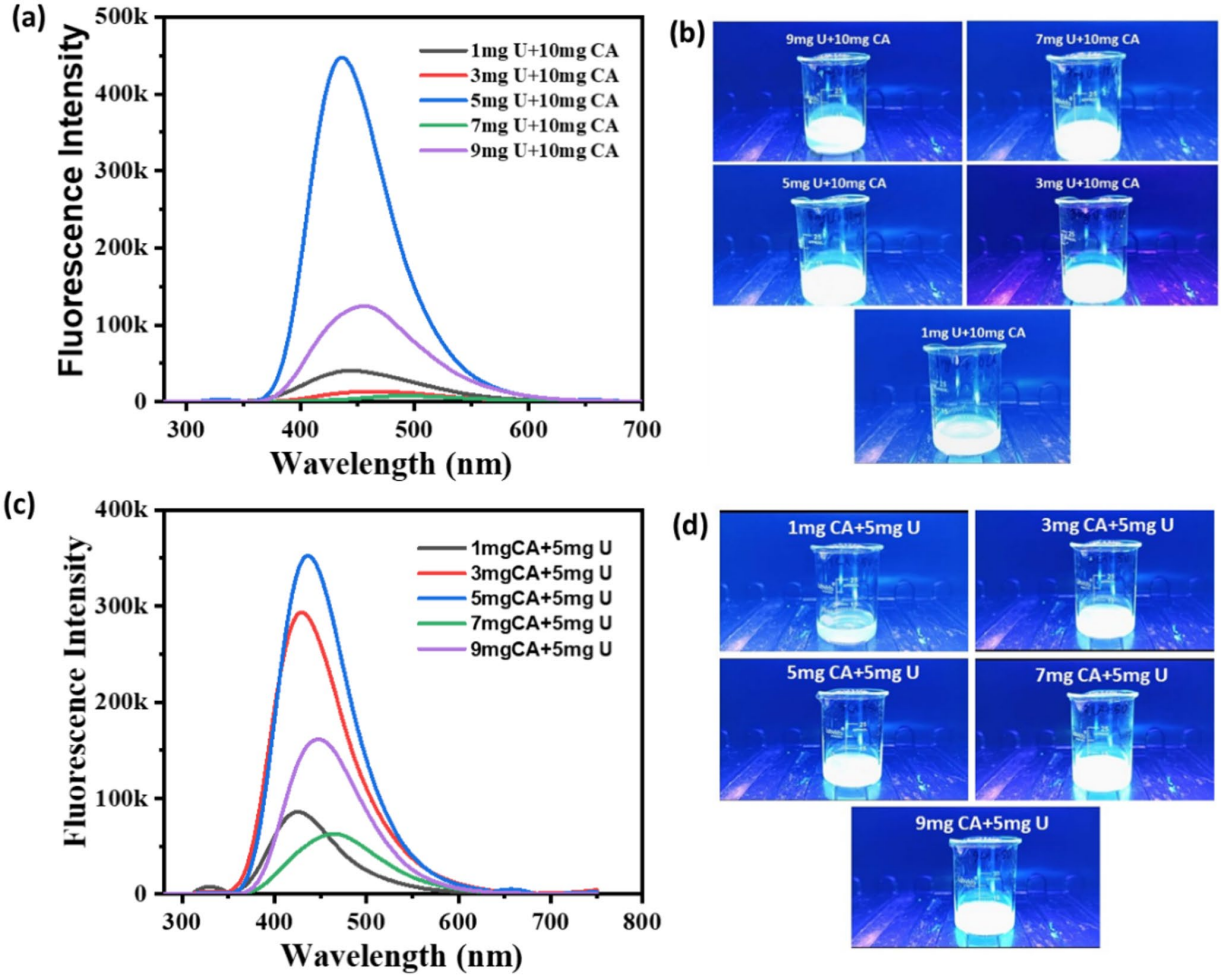
(**a**, **b**) Fluorescence spectra obtained by CDs formed from varying concentrations of urea in 10 mg citric acid and fluorescence image, respectively. (**c**, **d**) Fluorescence spectra obtained by CQDs formed from varying concentrations of citric acid in 5 mg urea and fluorescence image, respectively

The investigation of varying urea concentrations (1, 3, 5, 7, and 9 mg) alongside a fixed citric acid concentration (10 mg) indicates that peak fluorescence intensity is attained at 5 mg of urea, followed in decreasing order by 9 mg, 1 mg, 3 mg, and 7 mg, as shown in Fig. 2a. The lowest fluorescence intensity is recorded at 7 mg of urea. This observation implies that 5 mg is the optimal concentration of urea for the synthesis of carbon dots, as it yields the most favorable enhancement of fluorescence properties. Deviations from this concentration, whether in excess or deficiency, adversely influence fluorescence, potentially due to the emergence of surface defects or aggregation phenomena. The fluorescence image of the CDs prepared by varying the urea concentration is shown in Fig. 2b. Within an alternative experimental arrangement, varying concentrations of citric acid (1, 3, 5, 7, and 9 mg) were manipulated while urea remained at a steady 5 mg, revealing the highest fluorescence intensity at 5 mg of citric acid, with the following recorded amounts of 3 mg, 9 mg, 1 mg, and 7 mg, as shown in Fig. 2c. The minimum fluorescence was noted at 7 mg of citric acid. The fluorescence image of the CDs prepared by varying the citric acid concentration is shown in Fig. 2d. This finding substantiates that 5 mg of citric acid represents the optimal concentration, as it promotes the most effective fluorescence, whereas deviations from this concentration lead to quenching or structural modifications that impair emission efficiency.

**Fig. 3:**
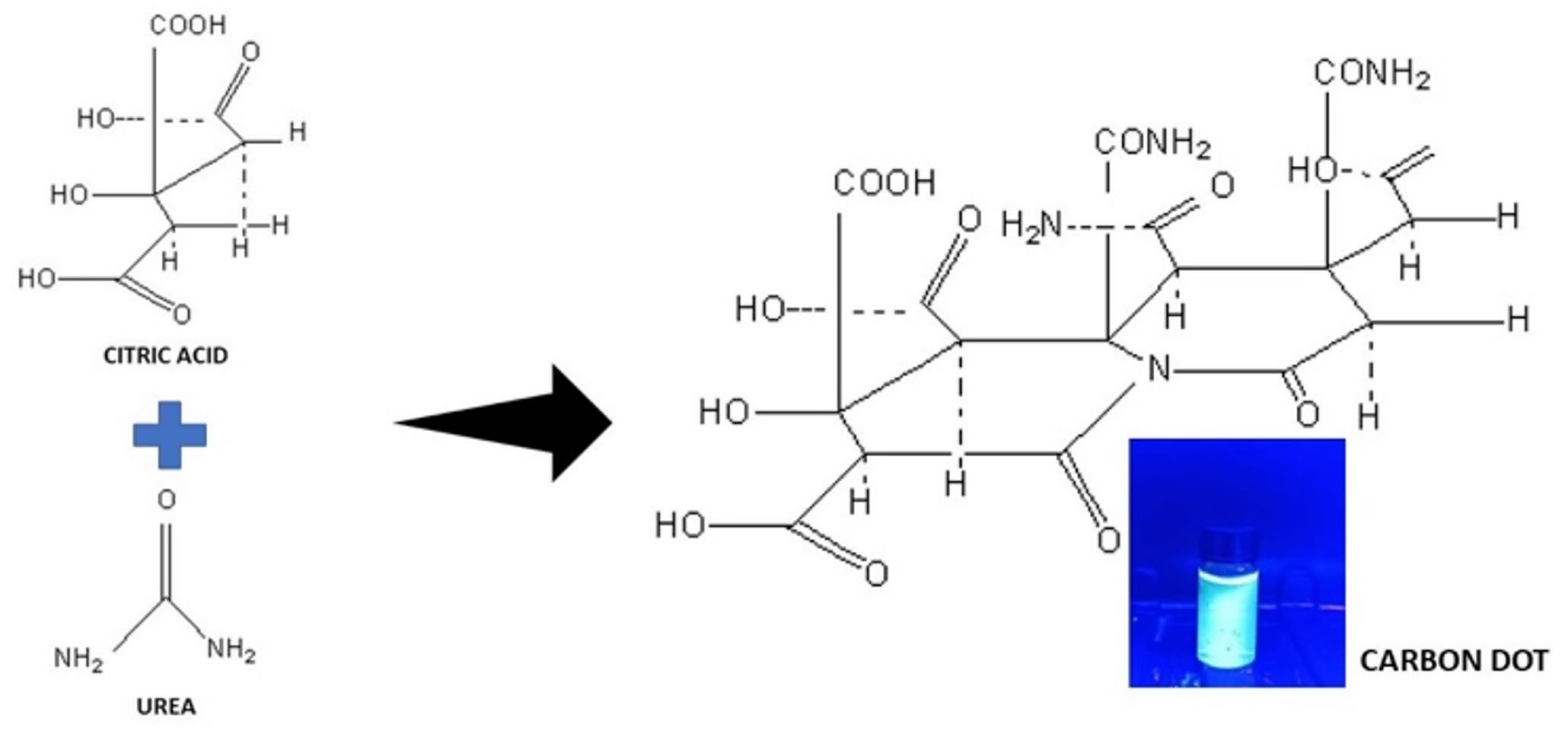
Schematic representation of carbonization of citric acid

Based on the data collected, the ideal parameters for synthesizing CDs are established at 5 mg for both urea and citric acid. For optimal fluorescence outcomes, the critical 1:1 stoichiometric ratio of urea and citric acid is essential, as it secures a balanced approach to nitrogen doping (from urea) and the sourcing of carbon precursors (from citric acid). Fig. 3 shows the carbonization of citric acid in the process of formation of CDs. The ratio 1:1 is essential for maximizing quantum yield and to produce high-quality carbon dots exhibiting enhanced optical properties.

### Optimization of Microwave Time

**Fig. 4:**
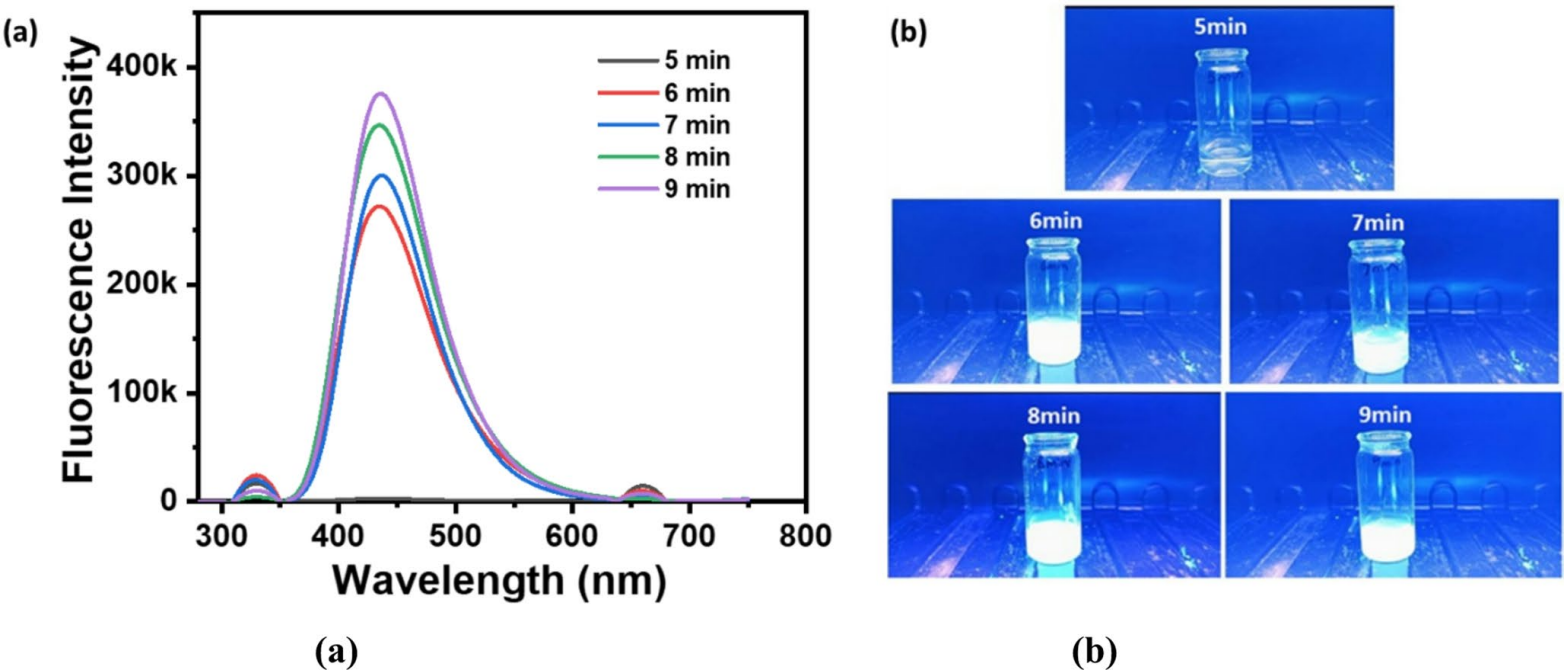
(**a**, **b**) Fluorescence spectra obtained by CDs prepared from 1:1 urea: citric acid at varying microwave exposure time and fluorescence image of the CDs, respectively

Fig. 4a shows the fluorescence intensity patterns of CDs synthesized by varying microwave reaction durations of 5, 6, 7, 8, and 9 min. The most pronounced fluorescence intensity is recorded at the 9-minute mark, succeeding in which the intensities at 8, 7, and 6 min are observed in decreasing order, whereas the fluorescence at 5 min is minimal. Fig. 4b shows the fluorescence image of the CDs synthesized by varying the time. This observation implies that the duration of synthesis exerts a significant effect on the optical characteristics of CDs. At reduced reaction durations (for instance, 5 min), the fluorescence is notably low, presumably attributable to incomplete carbonization and inadequate formation of the CDs architecture. With the extension of reaction time, the carbonization process advances, facilitating the development of well-defined CDs. The progressive increase in fluorescence intensity from 6 to 9 min indicates that prolonged reaction intervals enable more effective nitrogen doping.

### Detection of Urea in Soil

**Fig. 5:**
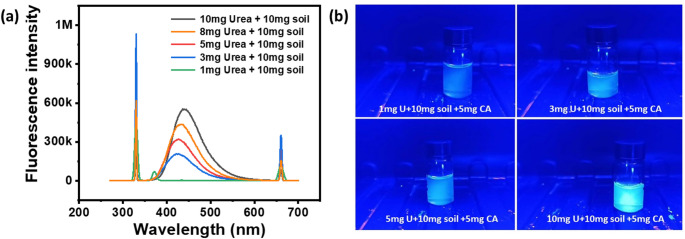
(**a**, **b**) Fluorescence and fluorescence spectra obtained by the presence of urea in the soil sample

The fluorescence plot in Fig. 5a illustrates the association between fluorescence intensity and wavelength for soil samples that are marked by various urea concentrations (1 mg, 3 mg, 5 mg, and 10 mg), with 5 mg of citric acid. An increase in fluorescence intensity is observed with the escalation of urea concentration in the soil, thereby signifying a proportional relationship between urea levels and the fluorescence signal by the interaction with citric acid.

The sample containing 10 mg of urea has high fluorescence intensity. This is subsequently followed by samples with 5 mg, 3 mg, and 1 mg of urea, which exhibit diminishing fluorescence intensities in alignment with their respective urea concentrations. The trend indicates that the incorporation of citric acid amplifies the detection of urea in the soil by generating a fluorescence signal that is directly correlated with the urea content. The fluorescence image of samples with varying concentrations of urea is shown in Fig. 5b. This methodology presents a viable analytical technique for quantitatively assessing urea in soil matrices. The elevated fluorescence intensity recorded for higher urea concentrations underscores the method’s sensitivity.

### Effect of pH

**Fig. 6:**
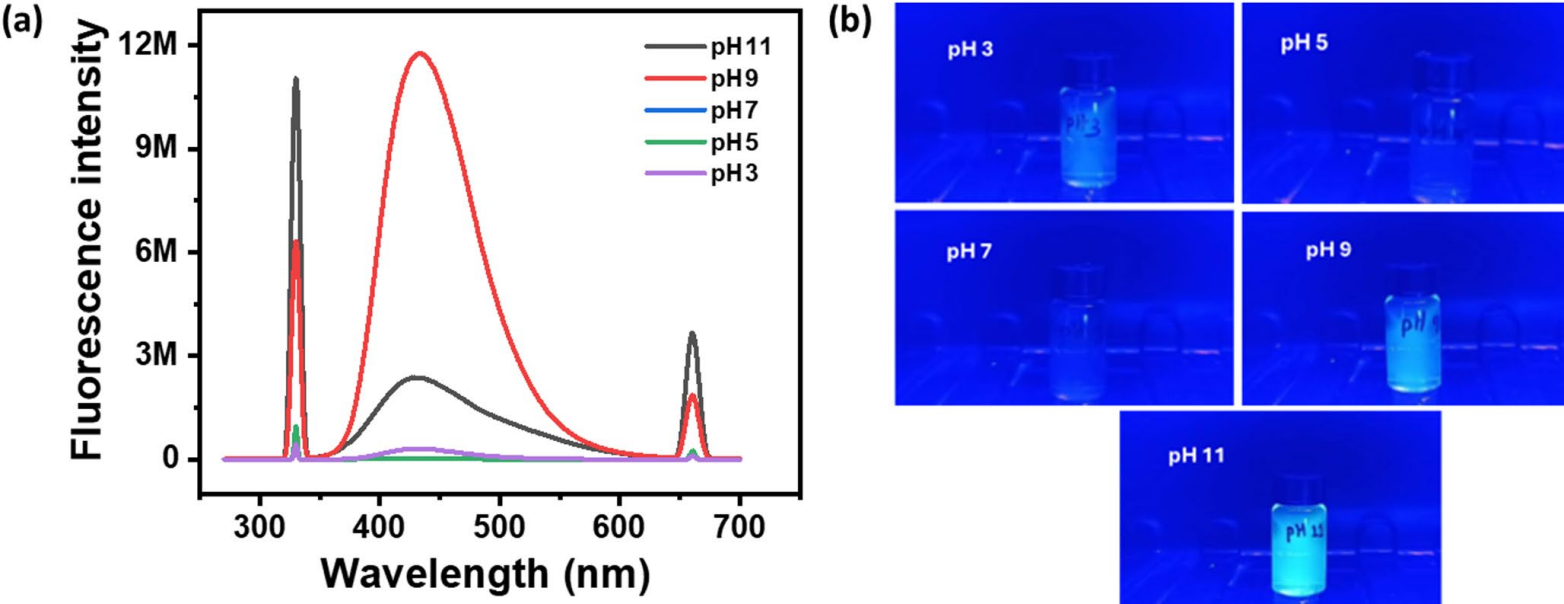
(**a**) Fluorescence spectra obtained in pH study. (**b**) Photograph of CDs fluorescence prepared at different pH

The fluorescence graph displays the emission intensity of CDs synthesized by urea and citric acid in the soil at varying pH values. Fluorescence is at its best in alkaline medium, as indicated by the highest intensity at pH 9, as shown in Fig. 6a. While pH 11 exhibits less fluorescence than pH 9, lower intensities are seen in acidic (pH 3 and pH 5) and neutral (pH 7) situations. The peaks (~ 450–500 nm) stay constant, indicating that pH variations do not affect the fluorescence process despite variations in intensity. The fluorescent image of the CDs prepared in different pH is shown in Fig. 6b. The pH study suggests that pH 9 is the best pH for urea detection in the soil since it is where the CD’s fluorescence intensity is the highest.

### Characterization of CDs

**Fig. 7:**
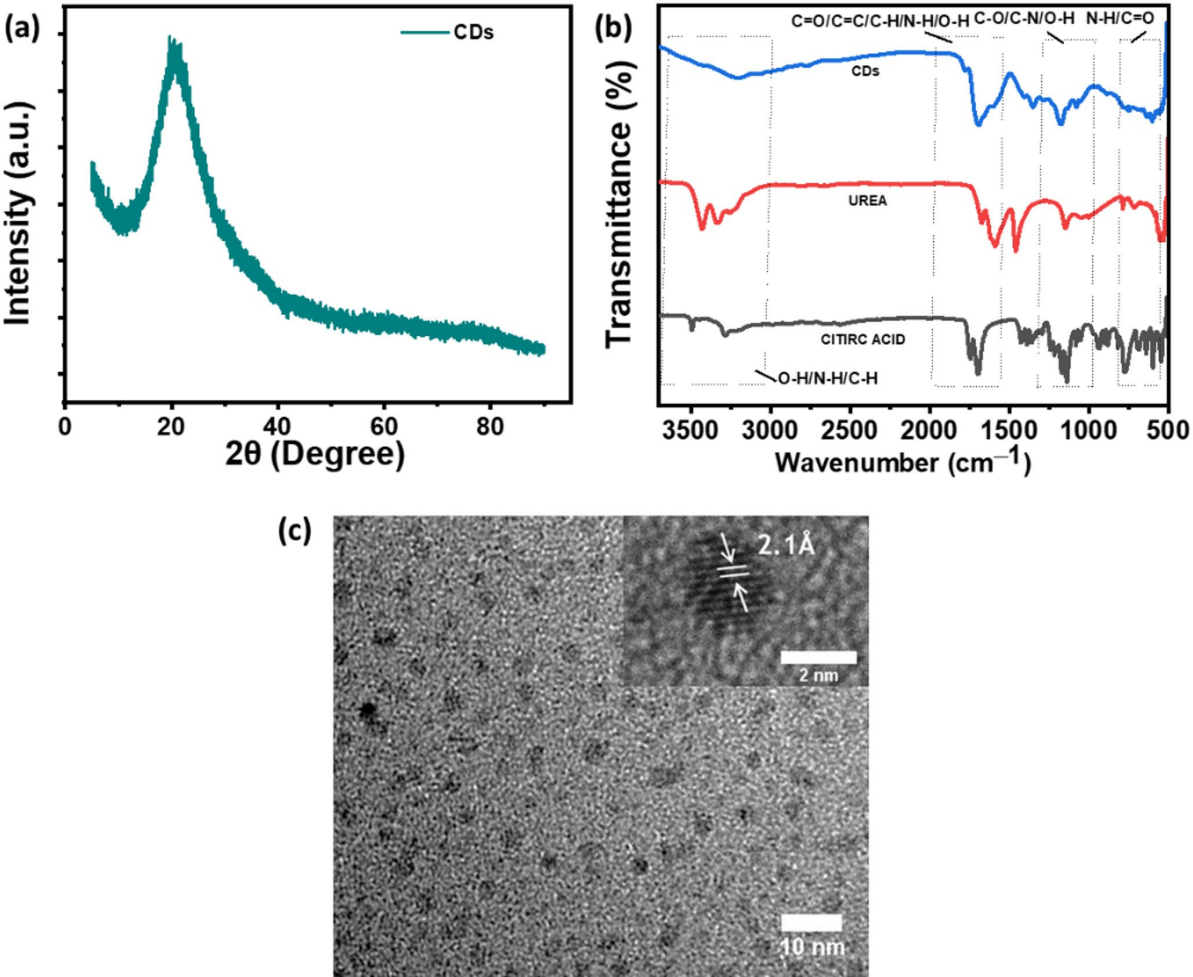
Characterization of CDs prepared from urea-to-citric acid weight ratio of 1:1, treated with microwave irradiation lasting 9 min. (**a**) XRD spectrum. (**b**) FTIR plot. (**c**) TEM image of CDs. Inset in the figure shows the high-resolution TEM image of a CD with d-spacing

The X-ray diffraction (XRD) assessment results illustrated in Fig. 7a demonstrate the formation of CDs at a urea-to-citric acid weight ratio of 1:1, treated with microwave irradiation lasting 9 min. The carbon content inherent in the CDs is evidenced by the emergence of a broad peak in the XRD profile at 2θ = 20.50°, which correlates with the (002) crystallographic plane [31]. The FTIR analysis in Fig. 7b elucidates the chemical transformations occurring during synthesizing CDs derived from citric acid and urea. The spectral profile of citric acid shows characteristic functionalities of carboxylic acids, which include a prominent C = O stretching vibration within the range of 1700–1750 cm⁻¹ and O–H bending vibrations observed between 1400 and 1450 cm⁻¹ [32]. In the spectrum of the CDs, the attenuation or complete absence of these peaks implies processes of condensation or cyclization. The spectral representation of urea exhibits diminished or displaced peaks corresponding to C = O stretching (~ 1650 cm⁻¹) and N–H bending (~ 1550–1600 cm⁻¹), which are indicative of amide functionalities [33]. The CDs display specific spectral properties, including a wide band between 3200 cm⁻¹ and 3600 cm⁻¹ associated with N–H and O–H vibrational modes, suggesting existence of hydrogen bonding interactions [34]. A novel peak observed around ~ 1600–1650 cm⁻¹ signifies C = C stretching, thereby corroborating the existence of aromatic or sp²-hybridized carbon structures [33, 35]. Furthermore, peaks located within the range of 1000–1300 cm⁻¹ are ascribed to C–O stretching vibrations. Spanning from 1500 to 500 cm⁻¹, the fingerprint zone highlights the presence of C–O and C–C bending pertinent to citric acid, coupled with C–N stretching (~ 1000–1250 cm⁻¹) and N–H wagging (~ 700–900 cm⁻¹) tied to urea [36]. Fig. 7b shows the TEM image of the CDs. The relatively uniform size and dispersion imply successful synthesis without significant aggregation. The inset at the top-right shows a zoomed-in view of a single CD, revealing lattice fringes. The measured interplanar spacing (d-spacing) was 2.1 Å, corresponding to the (100) plane of graphitic carbon.

**Fig. 8:**
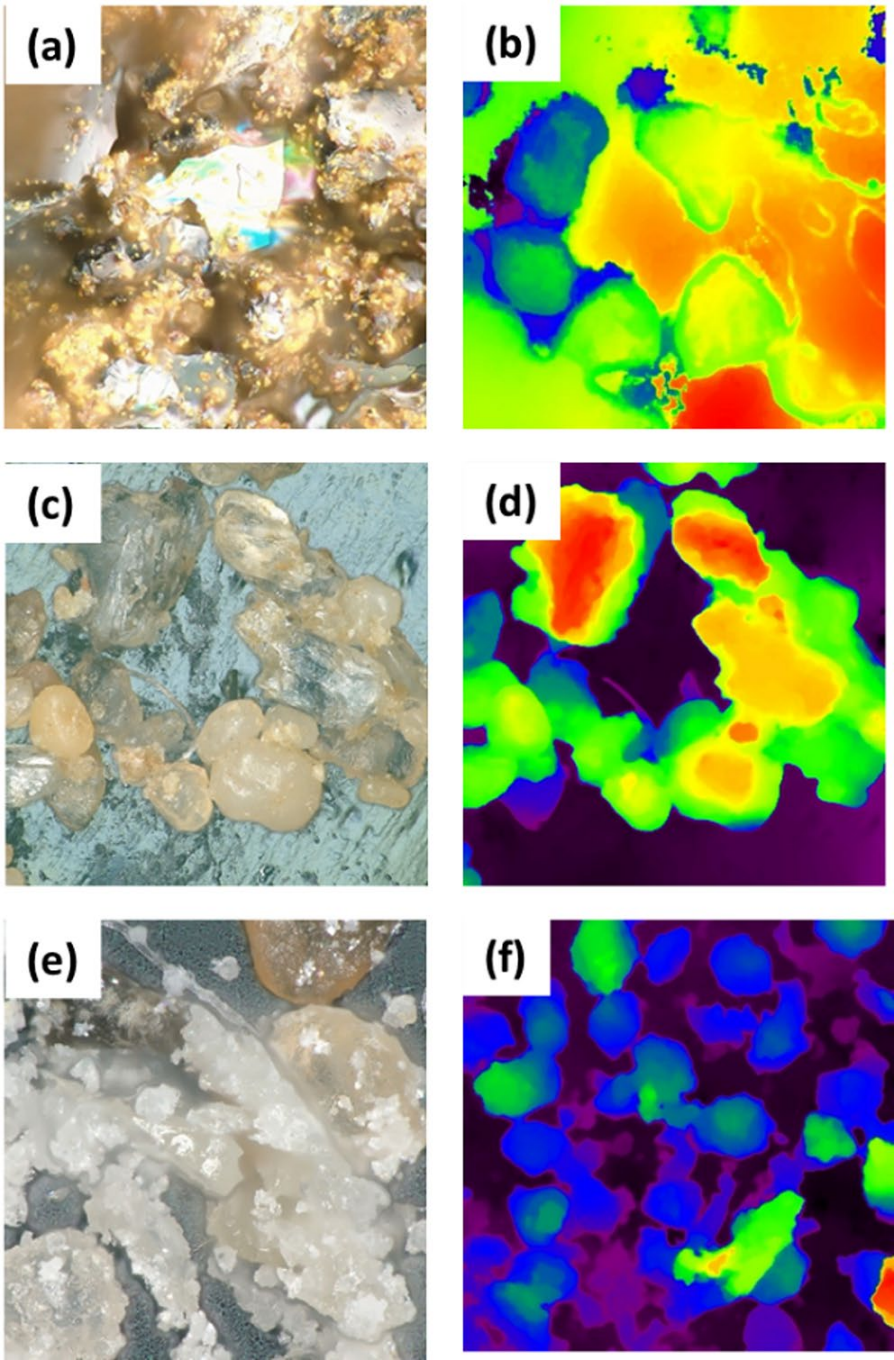
Profilometric examination. (**a**, **b**) CDs obtained from citric acid and urea. (**c**, **d**) soil sample containing urea. (**e**, **f**) soil sample with urea and citric acid after microwaving

Fig. 8 shows the optical profilometry characterization. The profilometric examination elucidates the microstructural and topographical attributes of a soil specimen containing urea crystals, the soil sample with urea and citric acid, CDs prepared from citric acid and urea. All the images have 500 μm scale bar. In the case of CDs synthesized via citric acid and urea, the chromatic representation (see Fig. 8a) illustrates heterogeneous, well-defined structures, whereas the height map (see Fig. 8b) indicates considerable height discrepancies attributable to nanoscale aggregation and functionalization processes. Within the soil-urea system (see Fig. 8c and d), urea crystals are seen as smooth, translucent entities unevenly distributed throughout the granular soil matrix. The height map delineates prominent peaks that correspond to urea crystals. The incorporation of citric acid into soil containing urea (see Fig. 8e and f) yields smoother and more homogenous surface characteristics. The color image illustrates finer, more evenly distributed particles, while the height map demonstrates a reduction in irregularities and a more consistent variation in height. Citric acid enhances the integration and stabilization of urea, thereby alleviating surface roughness.

**Fig. 9:**
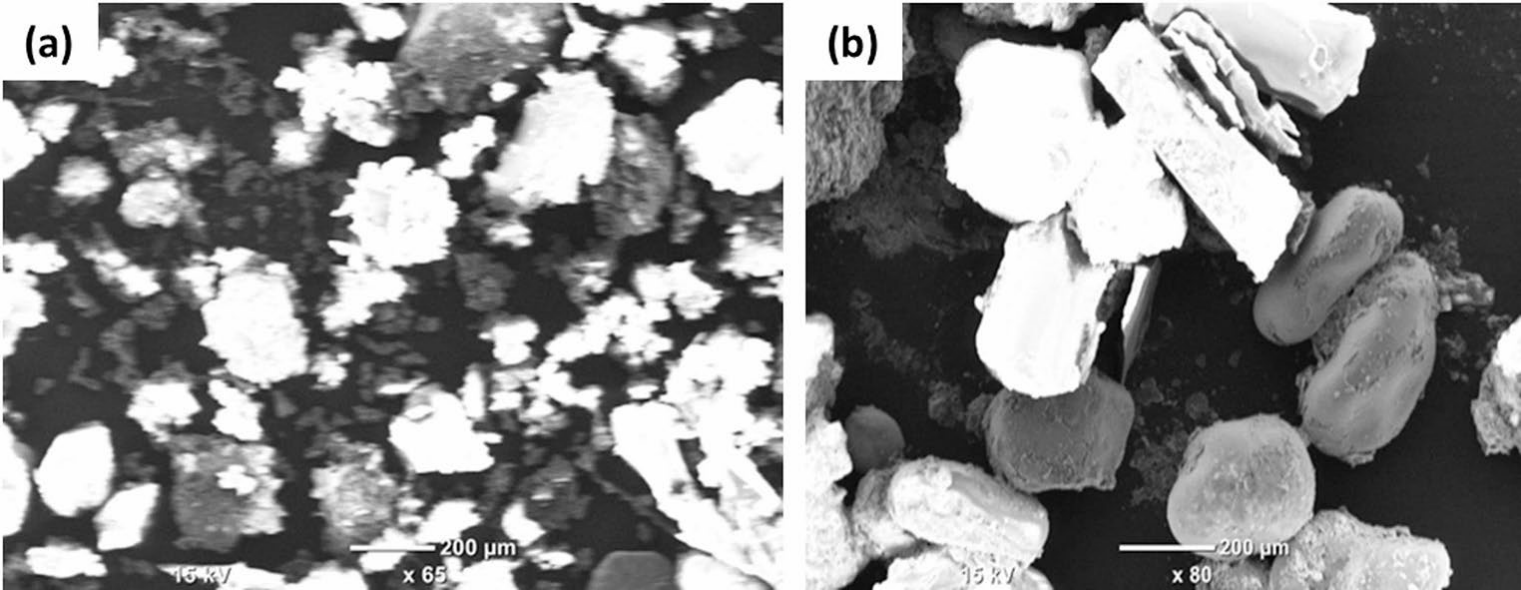
SEM images of (**a**) Soil with urea and citric acid (**b**) soil with urea

The SEM analysis was performed to provide a view of the microstructural aspects of soil blended with urea together with CDs, and soil with urea. Fig. 9a illustrates soil that integrates urea and CDs, wherein the CDs amalgamate with the soil matrix, facilitating finer surface characteristics and enhanced urea distribution. The urea crystals appear interspersed and less isolated, indicating augmented stabilization attributed to the presence of CDs. In Fig. 9b, the soil containing only urea reveals large, smooth, and distinctly defined urea crystals distributed unevenly, exhibiting minimal interaction with the granular soil matrix.

**Fig. 10:**
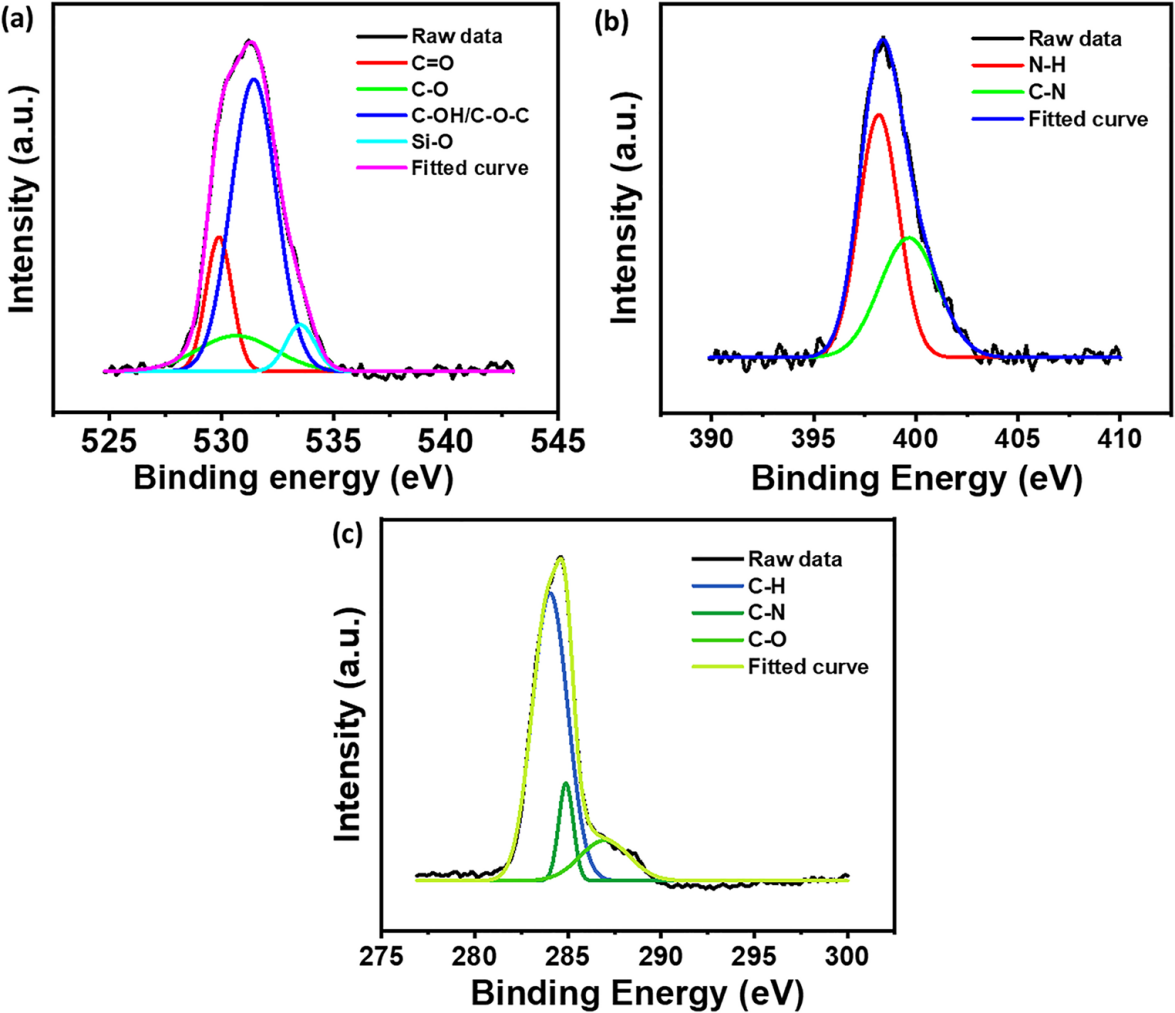
High-resolution XPS spectra (**a**) O1s (**b**) N1s (**c**) C1s

The soil samples containing urea and citric acid were subjected to XPS. Oxygen and nitrogen functional components are competently incorporated into carbon dots generated with citric acid and urea, according to detailed X-ray Photoelectron Spectroscopy (XPS) findings. The C = O, C‒OH, and C‒O‒C functionalities from citric acid are linked to peaks in the energy range of 530–535 eV that are visible in the O1s spectrum [37] (see Fig. 10a). These spectrum characteristics indicate oxygen functionalities, which are most likely produced by condensation or esterification procedures. Peaks at 399–401 eV in the N1s spectra (see Fig. 10b) are attributed to N–H and C–N bonding [19]. While the C-N peak confirms the presence of nitrogen in the carbon matrix, most likely in the form of graphitic or pyridinic nitrogen, the N-H signal indicates the presence of amine groups produced from urea. These nitrogenous species enhance the CDs’ chemical and electrical properties. The C1s spectra (see Fig. 10c) show peaks that correspond to C-H, C-O, and C-N bonds and are located between 284 eV and 288 eV [19]. While the peaks at higher binding energies verify the presence of oxidised carbon and nitrogen interactions, the peak at about 284 eV is associated with sp^2^/sp^3^ hybridized carbon [37].

### Limit of Detection

**Fig. 11:**
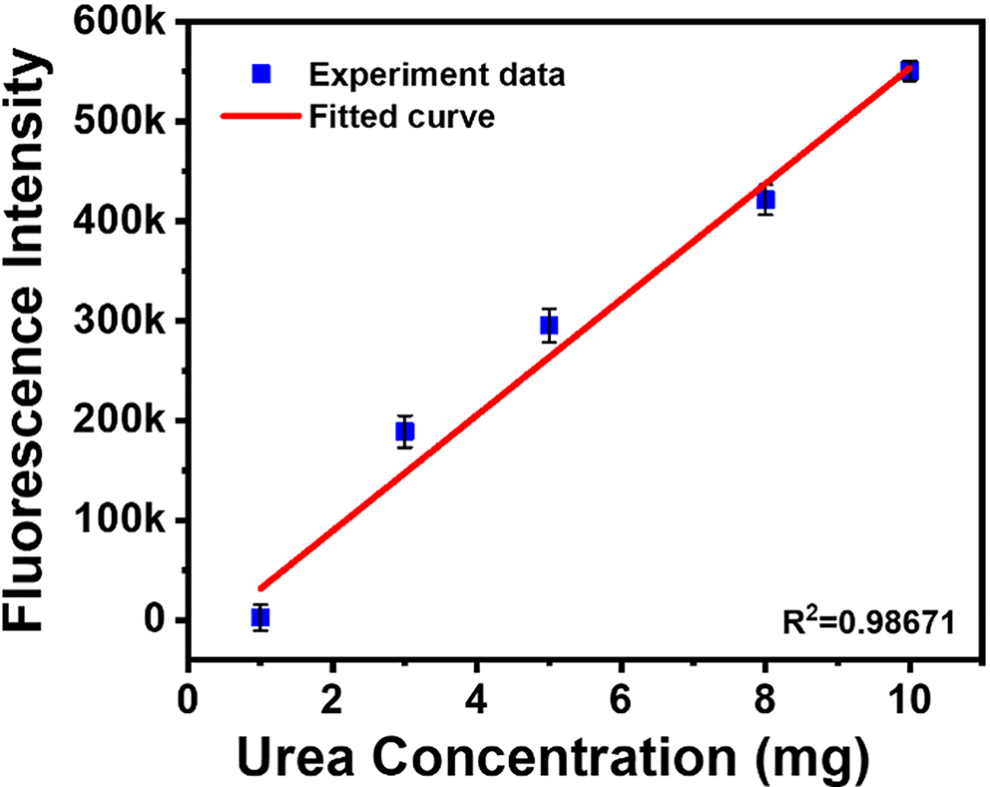
Calibration plot for urea detection

Fig. 11 shows the calibration graph correlating fluorescence intensity and concentration of urea by examining the obtained peak fluorescent intensity. The calibration curve was plotted by considering the fluorescence intensity at the wavelength of 439 nm under varying urea concentration, as shown in Fig. 5a A linear fit to the correlation was performed to determine the limit of detection (LOD) based on the 3σ/m standard, where m indicates the slope of the calibration graph, and σ represents the standard deviation of the intercept. The linear fit is illustrated in Fig. 11. As the soil concentration was kept constant (10 mg) and urea concentration was varied (see Fig. 5a), the LOD was calculated to be approximately 143 mg/g of soil concentration with R^2^ = 0.98.

### Selectivity Analysis

The selectivity of the synthesized carbon dots (CDs) for the detection of urea was assessed by conducting a comparative analysis of their fluorescence responses in the presence of various potentially interfering metal ions, such as Cu²⁺, Fe³⁺, Mg²⁺, Pb²⁺, and Zn²⁺. The fluorescence intensity of the CDs was meticulously recorded across a spectrum of wavelengths to determine the presence of any significant interference.

**Fig. 12:**
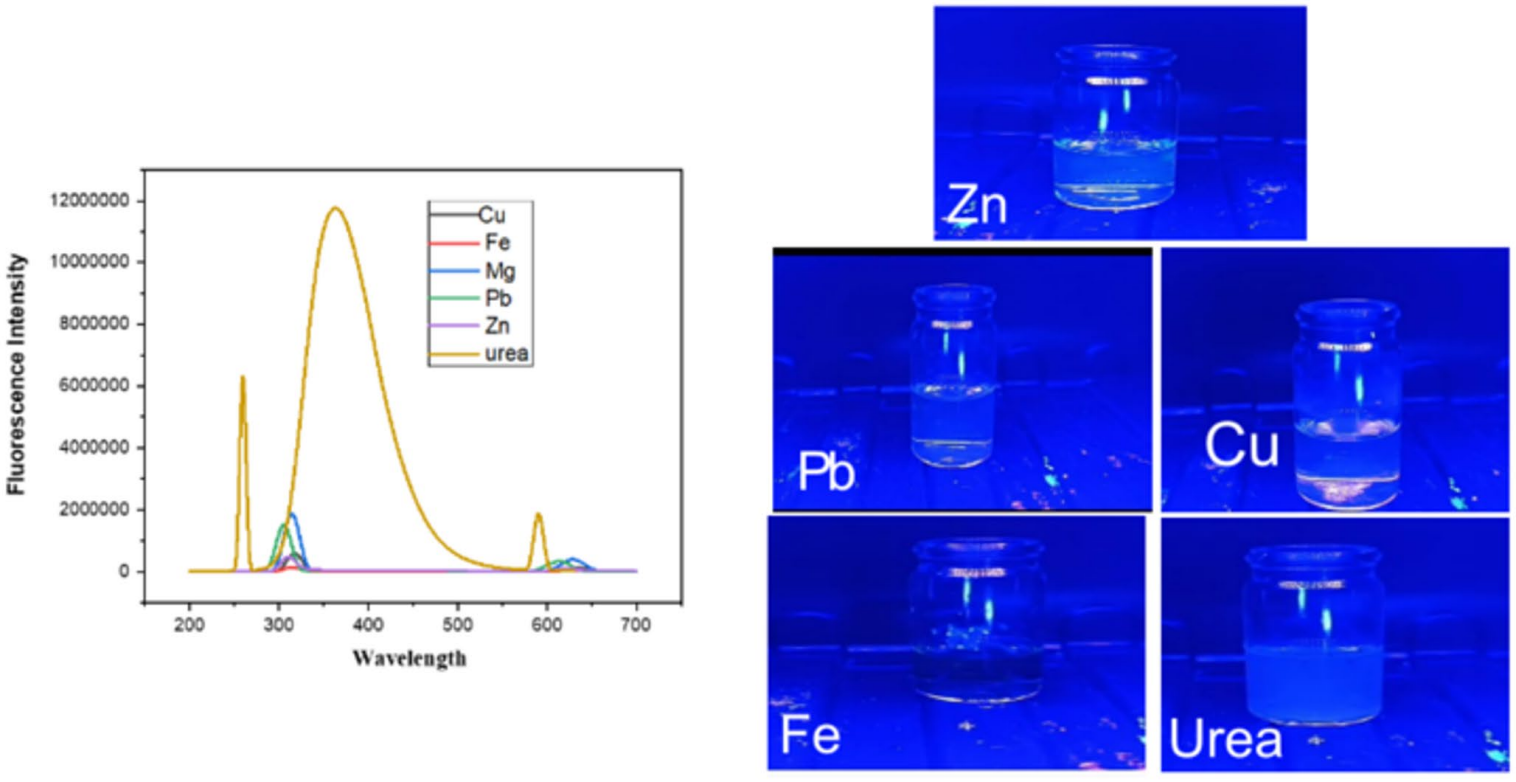
Fluorescence spectra and fluorescence obtained in selectivity analysis using metal ions such as zinc, lead, copper, and iron when compared with urea

As illustrated in the fluorescence spectra (see Fig. 12), urea demonstrates a pronounced fluorescence response, whereas the other metal ions exhibit minimal or negligible fluorescence intensity. The markedly elevated fluorescence intensity observed for urea substantiates the enhanced selectivity of the CDs towards urea molecules when juxtaposed with metal ions, which could potentially obfuscate detection. The findings suggest that the CDs engage specifically with urea, resulting in an augmented fluorescence signal. At the same time, the presence of metal ions does not induce any considerable fluorescence quenching or enhancement.

Overall, the study presents fluorescence-based detection of urea in the soil. Under microwave irradiation, citric acid has a higher reactivity towards urea present in the soil compared to other native inorganic compounds in the soil. However, the contribution from the native organic compounds in the highly organic soil samples could potentially interfere with the fluorescence intensity. To minimize such effects, the soil samples could be standardized before the testing by a pre-treatment step. Future work will focus on soil sample standardization to reduce native organics and study urea detection using the present method in diverse soil types.

## Conclusions

This study demonstrates a fluorescence-based method for identifying urea in the soil. A green microwave-assisted process was adopted for synthesizing nitrogen-doped carbon dots (CDs). The study identified citric acid and urea as the key precursors, with a vital 1:1 weight ratio and 9-minute microwave irradiation to enhance fluorescence features. Characterization assessments confirmed the structural and optical fidelity of the synthesized carbon dots, while fluorescence examinations underscored their robust responsiveness to varying urea concentrations and pH levels, particularly within slightly alkaline conditions. The proposed technique demonstrated remarkable sensitivity, with a detection limit established at 143 mg/gm for urea detection in soil. The successful implementation of this methodology on soil samples further substantiated its feasibility for agricultural monitoring, facilitating accurate urea quantification within complex matrices. This research bridges the divide between environmental sustainability and analytical accuracy by employing principles of green chemistry and utilizing cost-effective materials. It presents a scalable and environmentally benign solution to the challenges inherent in nitrogen management, which is vital for enhancing agricultural productivity and alleviating environmental repercussions. Subsequent investigations could build upon these discoveries by investigating additional functionalization techniques for CDs to augment selectivity and expand the methodology’s applicability across a variety of environmental and biological contexts.

## Data Availability

Data that supports the finding of this study is provided within the manuscript.
